# Measurement of disordered eating in bariatric surgery candidates: a systematic review of the literature

**DOI:** 10.1186/2050-2974-1-S1-O22

**Published:** 2013-11-14

**Authors:** Katrina Parker, Leah Brennan

**Affiliations:** 1 Centre for Obesity Research and Education (CORE), Monash University. School of Psychology and Psychiatry, Monash University, Australia

## 

Symptoms of disordered eating are common among patients seeking bariatric surgery, and assessment of eating pathology is typical in pre-surgical evaluations. This systematic review evaluates the methods used to assess disordered eating in bariatric surgery candidates. A systematic literature search of papers published from the emergence of bariatric surgery in the literature to March 2012 was conducted to identify original studies measuring eating psychopathology in adults seeking bariatric surgery. One hundred and forty-seven articles were identified, which featured 34 different questionnaires and 45 different interviews used in assessments prior to surgery. Limitations of the literature reviewed included the large variety and application of measures, limited description of assessments, and inconsistency in eating disorder criteria. Results demonstrate a lack of measures designed specifically for an obese sample and limited psychometric evaluation of measures in bariatric surgery candidates. The psychometric data available suggest that interview assessments are critical for accurately identifying binge episodes and diagnostic information, while self-report questionnaires may be beneficial for screening and providing additional information of clinical utility (e.g., eating, shape and weight-related cognitions). The use of validated measures will ensure accurate identification of disordered eating in the pre-surgical population, enabling appropriate targeting of intervention to optimise treatment success.

This abstract was presented in the **Disordered Eating – Characteristics & Treatment** stream of the 2013 ANZAED Conference.

